# Forsythoside A attenuates metabolic dysfunction in type 2 diabetic mice by inhibiting the MAPK and activating the Nrf2 signalling pathways

**DOI:** 10.2478/aiht-2026-77-4026

**Published:** 2026-03-30

**Authors:** Mengxian Shu, Chunhui Xiang

**Affiliations:** Central Hospital of Enshi Tujia and Miao Autonomous Prefecture, Department of Pulmonology and Endocrinology, Enshi City, China; Central Hospital of Enshi Tujia and Miao Autonomous Prefecture, Department of Neurosurgery, Enshi City, China

**Keywords:** body weight, catalase, cholesterol, glucose, glutathione peroxidase, HO-1, insulin, malondialdehyde, metformin, reactive oxygen species, superoxide dismutase, triglycerides, glukoza, glutation-peroksidaza, HO-1, inzulin, katalaza, kolesterol, malondialdehid, metformin, reaktivne kisikove vrste, superoksid-dismutaza, tjelesna masa, trigliceridi

## Abstract

Forsythoside A, a natural phenylethanoid glycoside extracted from the weeping forsythia (*Forsythia suspensa*), exhibits a wide range of pharmacological activity, including antibacterial, hepatoprotective, antioxidant, neuroprotective, antiviral, and anti-inflammatory. The aim of this study was to determine its anti-hyperglycaemic and antioxidative effects in a diabetic mouse model (created by administering high-fat diet alongside successive low doses of streptozotocin) by measuring fasting blood glucose levels, body weight, food and water intake, oxidative stress, and histopathological changes. Diabetic mice received either forsythoside A (30 or 60 mg/kg bw) or metformin (150 mg/kg) as standard type 2 diabetes medication for comparison. After four weeks of administration, forsythoside A significantly increased body weight and reduced food and water intake at both doses, while the higher, 60 mg/kg dose also significantly reduced fasting blood glucose and had a similar effect on all these parameters as metformin. The higher, 60 mg/kg dose also had similar antioxidative effects as metformin in lowering malondialdehyde (MDA) and reactive oxygen species (ROS) and in elevating the antioxidant superoxide dismutase (SOD), glutathione peroxidase (GSH-Px), and catalase (CAT) levels. Moreover, at 60 mg/kg forsythoside A attenuated lipid accumulation in diabetic mice by elevating high-density lipoprotein cholesterol (HDL-C) and lowering total cholesterol (TC), triglycerides (TG), and low-density lipoprotein cholesterol (LDL-C), showing comparable effect to metformin. Similar improvements were observed by histopathological changes in the liver. Forsythoside A also lowered insulin levels in diabetic mice by up-regulating p-IRS-1 and inhibited the mitogen-activated protein kinase (MAPK) pathway by lowering the expressions of the p-p38 and p-JNK proteins. At the same time, it promoted the Nrf2 pathway by increasing Nrf2 and HO-1 expressions relative to untreated diabetic mice. In conclusion, forsythoside A demonstrated therapeutic effects akin to those of 150 mg/kg metformin and may be a promising candidate for clinical application.

## Abstract (hr)

Forsitozid A prirodni je feniletanoidni glikozid izoliran iz viseće forzicije (*Forsythia suspensa*) sa širokim rasponom farmakoloških djelovanja, uključujući antibakterijsko, hepatoprotektivno, antioksidacijsko, neuroprotektivno, antivirusno i protuupalno djelovanje. Cilj ovoga istraživanja bio je utvrditi njegov antihiperglikemijski i antioksidacijski učinak u mišjem modelu dijabetesa (induciranom primjenom visokomasne prehrane uz uzastopne niske doze streptozotocina) mjerenjem razine glukoze u krvi natašte, tjelesne mase, unosa hrane i vode, pokazatelja oksidacijskoga stresa te uvidom u histopatologiju jetre. Dijabetični su miševi dobivali ili forsitozid A (30 ili 60 mg/kg tjelesne mase) ili, za usporedbu, metformin (150 mg/kg) kao standardni lijek za dijabetes tipa 2. Nakon četiri tjedna primjene, forsitozid A značajno je pri objema dozama povećao tjelesnu masu te smanjio unos hrane i vode, a viša doza od 60 mg/kg također je značajno snizila razinu glukoze u krvi natašte i pokazala sličan učinak na sve navedene parametre kao metformin. Doza od 60 mg/kg pokazala je i usporedive antioksidacijske učinke s metforminom, smanjivši razine malondialdehida (MDA) i reaktivnih kisikovih vrsta (ROS) te povećavši razine antioksidacijskih enzima superoksid-dismutaze (SOD), glutation-peroksidaze (GSH-Px) i katalaze (CAT). Nadalje, forsitozid A u dozi od 60 mg/kg smanjio je nakupljanje lipida u dijabetičnih miševa povećanjem koncentracije lipoproteinskoga kolesterola visoke gustoće (HDL-C) te sniženjem ukupnoga kolesterola (TC), triglicerida (TG) i lipoproteinskoga kolesterola niske gustoće (LDL-C), iskazujući učinak usporediv s metforminom. Slična poboljšanja potvrđena su i histopatološkim promjenama u jetri. Forsitozid A također je snizio razinu inzulina u dijabetičnih miševa pojačanom ekspresijom p-IRS-1 te inhibirao signalni put mitogenom aktivirane protein-kinaze (MAPK) smanjenjem ekspresije proteina p-p38 i p-JNK. Istodobno je potaknuo signalni put Nrf2 povećanjem ekspresije Nrf2 i HO-1 u odnosu na netretirane dijabetične miševe. Naše istraživanje pokazalo je da su učinci forsitozida A usporedivi s onima metformina u dozi od 150 mg/kg, što upućuje na njegovu visoku učinkovitost u kontekstu moguće buduće kliničke primjene.

Type 2 diabetes mellitus (T2DM), with its 90 % prevalence in diabetic patients, is a serious, life-threatening chronic disease characterised by high blood glucose levels and metabolic disorders such as insulin resistance and hyperlipidaemia, which can lead to complications such as diabetic nephropathy, cardiopathy, and retinopathy (1).

Current therapy options include insulin and synthetic drugs such as metformin, rosiglitazone, and pioglitazone to improve glucose uptake and reduce its hepatic output (2), but these drugs may cause specific adverse effects, including weight gain, gastrointestinal discomfort, diarrhoea, nausea, and vomiting. Traditional Chinese medicines complementing antidiabetic medications have been shown to relieve these side effects (3), most notably the *Forsythiae Fructus*, the dried fruit of the weeping forsythia [*Forsythia suspensa* (Thunb.) Vahl] known for its antiviral, antibacterial, antioxidative, anti-inflammatory, antihyperlipidaemic, neuroprotective, and antidiabetic properties owed to the action of its phenylethanoid glycoside forsythoside A (Figure 1) (4,5,6,7,8,9). However, much is still unknown about the mechanisms of action that lead to the pharmacological effects of forsythoside A against T2DM. Of the many signalling pathways involved in T2DM pathogenesis (10), mitogen-activated protein kinase (MAPK) caught our attention, as it regulates glucose and lipid metabolism in the liver (11) by inhibiting the phosphorylation of insulin receptor substrate-1 (IRS-1), resulting in insulin resistance (12), and by down-regulating GLUT4 expression, which results in lower reduced glucose transport from blood (13). Furthermore, a study by Quan et al. (8) has demonstrated that T2DM can be alleviated by MAPK inhibition.

**Figure 1:**
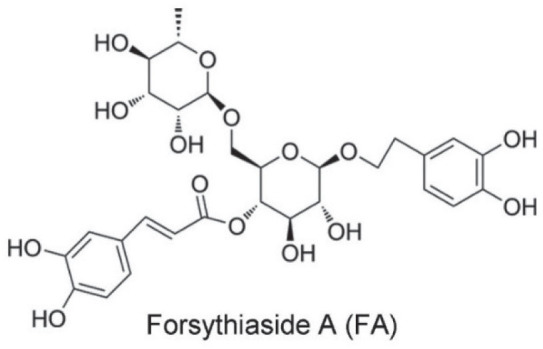
Chemical structure of forsythiaside A

The aim of our study was therefore to verify the effects of forsythoside A on blood glucose, serum insulin, oxidative stress, lipid accumulation, and hepatic damage in diabetic mice and see how they are related to the MAPK and nuclear factor erythroid2-related factor 2 (Nrf2) signalling pathways. We also wanted to see how it compares to metformin as the standard antidiabetic treatment.

## MATERIALS AND METHODS

### Mice model

The study involved 50 male C57BL/6J mice (20±2 g and 6–8 weeks old) obtained from Weitonglihua Biotechnology (Beijing, China) and kept in standard conditions (22–26 °C, 30–70 % relative humidity, 12/12 h light/dark cycle), as described in detail elsewhere (14). The experiments were approved by the Ethics Committee of the Central Hospital of Enshi Tujia and Miao Autonomous Prefecture and conducted in compliance with the National Institutes of Health Laboratory Animal Care and Use Guidelines.

Ten mice (n=10) served as control and were receiving normal diet, while the mice in the remaining four groups (n=10 each) were receiving high-fat diet (60 % fat, 20 % carbohydrate, 20 % protein; Weitonglihua Biotechnology) to induce diabetes as described elsewhere (15). After four weeks of such feeding regime, for four consecutive days, all but control mice were given 10 mL intraperitoneal injections of 30 mg/kg streptozotocin (Sigma-Aldrich, St. Louis, MO, USA) dissolved in citric acid (pH 4.5) for four consecutive days to induce diabetes. Control mice also received citric acid buffer over the same time to match exclude potential physiological confounders due to injection to diabetic mice. One week after receiving the last dose of treatment, we measured fasting blood glucose level in the tail vein of each mouse using an automated glucose monitor (HemoCue™ Glucose 201 DM, cat. No. 22601009, Thermo Fisher, Waltham, MA, USA) to see if they met the 16.7 mmol/L threshold for blood glucose to be considered diabetic. Having established diabetes in 36 of the 40 mice on high-fat diet and streptozotocin, we randomised them into four groups besides control, namely untreated diabetic mice (DM; n=10), those receiving 150 mg/kg bw metformin (MET; n=10), those receiving 30 mg/kg bw forsythoside A (FA 30; n=10), and those receiving 60 mg/kg bw forsythoside A (FA 60; n=6). The choice of the doses was based on previous research by Matsui et al. (16) on metformin and by Song et al. (17) on forsythoside A.

Metformin (Sigma-Aldrich) was given by oral gavage and either forsythoside A (Sigma-Aldrich) dose by intraperitoneal injection, all twice daily at 12-hour intervals (08:00 and 20:00 h) for another four weeks. Before injection, monomeric forsythoside A was dissolved in sterile saline (0.9 % NaCl) and prepared to the required concentration (30 mg/kg or 60 mg/kg bw) in the volume of 10 mL per injection.

The body weight of each mouse was recorded every week. Fasting blood glucose and food and water intake were also recorded at the end of the four-week treatment. To eliminate observation bias, the same researcher checked on the mice every day for any changes in weight and behaviour as well as for respiration, hunched posture, fur condition, spontaneous movement, and any symptoms of poisoning. No unexpected adverse occurrences or deaths were recorded over the four weeks of treatment.

### Serum and liver parameter measurements

After four weeks of treatment, the mice were sacrificed under sevoflurane anaesthesia and their blood samples taken from the tail vein and stored in tubes containing ethylenediaminetetraacetic acid (EDTA) to prevent coagulation. Serum was extracted by centrifugation at 3000 *g* for 10 min and serum insulin, total cholesterol (TC), triglycerides (TG), low-density lipoprotein cholesterol (LDL-C), and high-density lipoprotein cholesterol (HDL-C) measured using respective commercial kits (Abcam, Cambridge, UK) as described elsewhere (18).

Liver tissues were lysed in the radioimmunoprecipitation assay (RIPA) buffer (Beyotime, Beijing, China) supplemented with Phosphatase Inhibitor Cocktail 2 (Sigma-Aldrich). Tissues were centrifuged at 12000 *g* for 60 min and the levels of superoxide dismutase (SOD), glutathione peroxidase (GSH-Px), catalase (CAT), and malondialdehyde (MDA) determined in the supernatant using commercial kits (Abcam, Cambridge, UK) as described elsewhere (19).

## ROS measurements

Liver tissues were harvested immediately after sacrifice, embedded in optimal cutting temperature (OCT) medium (Beyotime, Beijing, China), and rapidly frozen with liquid nitrogen. Upon thawing, the tissues were cut into 8 µm slices, fixed with 4 % paraformaldehyde, and incubated in dark with 10 µmol/L 2′,7′-dichlorodihydrofluorescein diacetate (DCFH-DA) at 37 °C for 30 min. After washing with phosphate buffer saline (PBS), the slices were immediately observed and imaged under a fluorescence microscope (200× magnification, Axioscope A1, Dresden, Germany). For quantitative fluorescence analysis we used the ImageJ software.

### Histopathological examination

The liver slices, obtained as described above, were incubated with haematoxylin, treated with eosin (both obtained from Sigma-Aldrich), and photographed with an Olympus microscope (OME-9000, Olympus, Tokyo, Japan). To determine hepatic glycogen, the slices were also stained with periodic acid and Schiff (PAS) and photographed with the microscope under 200× magnification.

### Western blotting

Proteins were separated from liver tissue supernatant using sodium dodecyl-sulphate polyacrylamide gel electrophoresis (SDS-PAGE) and electro-transferred onto a polyvinylidene difluoride (PVDF) membranes blocked with 5 % skim milk, and probed with primary antibodies, all purchased from Abcam: anti-p-IRS-1 and anti-IRS-1 (1:2000), anti-p-p38 and anti-p38 (1:3000), anti-p-JNK and anti-JNK (1:4000), anti-Nrf2 and anti-HO-1 (1:5000), and anti-β-actin (1:6000). The membranes were washed with PBST and then incubated with horseradish peroxidase-labelled secondary antibody (1:5000) and visualised using enhanced chemiluminescence substrate kit (KGC4601-100, KeyGen Biotech, Jiangsu, China) to detect immunoreactivities.

### Statistical analysis

Data of at least three independent experiments are expressed as means ± standard errors of the mean (SEM) and analysed with Student’s *t*-test or one-way analysis of variance (ANOVA) using the GraphPad Prism software (GraphPad Software, Inc., San Diego, CA, USA). Statistical significance was set to p<0.05.

## RESULTS AND DISCUSSION

**Figure 2:**
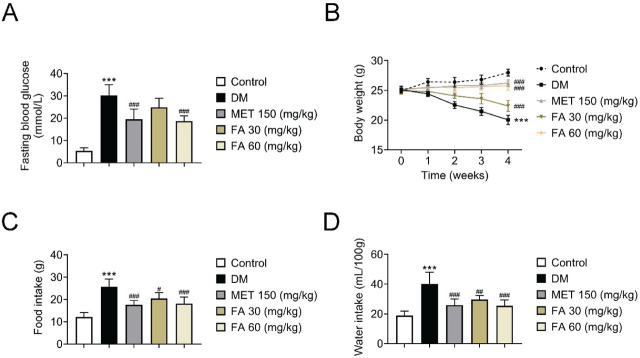
Effects of forsythoside A administration on: A) fasting blood glucose; B) body weight; C) food intake; and D) water intake in diabetic mice. *** *p*<0.001 vs control. # *p<*0.05; ## *p*<0.01; ### *p*<0.001 vs DM. DM – diabetic mice; FA – forsythoside A; MET – metformin

The antidiabetic effects of metformin and forsythoside did not differ significantly. Figure 2 compares the effects of forsythoside A administration on blood glucose, body weight, and food and water intake between the study groups. As expected, the diabetic mice (on high-fat diet plus streptozotocin regime) had the highest blood glucose levels and feed/water intake and the lowest body weight. Metformin significantly lowered fasting blood glucose and feed/water intake and increased body weight, as did the higher forsythoside A dose (60 mg/kg bw). The lower, 30 mg/kg bw forsythoside A dose yielded weaker but still significant effects, save for blood glucose.

**Figure 3:**
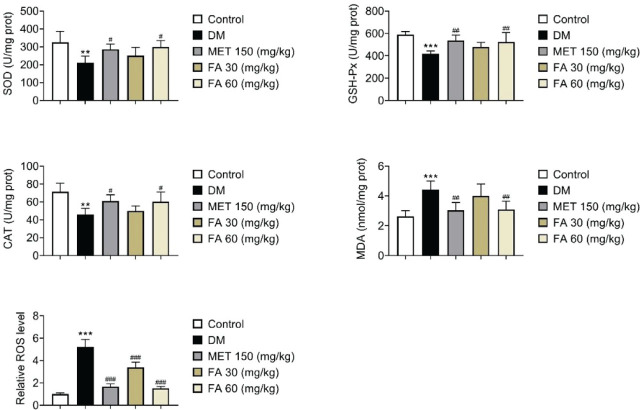
Effects of forsythoside A administration on oxidative stress parameters in diabetic mice. ** *p*<0.01; *** *p*<0.001 vs control. # *p<*0.05, ## *p*<0.01, ### *p*<0.001 vs DM. DM – diabetic mice; FA – forsythoside A; MET – metformin

Similarly, the higher forsythoside A dose significantly improved antioxidant levels (SOD, CAT, and GSH-Px) and lowered oxidative stress (MDA and ROS) in the liver tissue of diabetic mice with a similar effectiveness as metformin (Figure 3).

**Figure 4:**
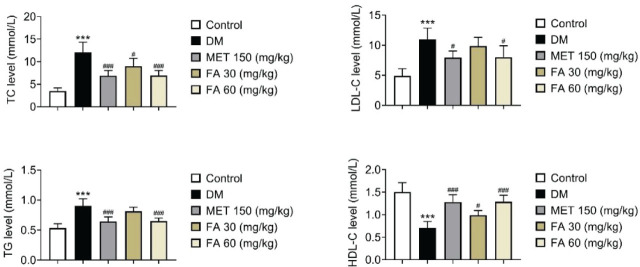
Changes in lipid accumulation in diabetic mice treated with forsythoside A. *** *p*<0.001 vs control. # *p<*0.05; ### *p*<0.001 vs DM. DM – diabetic mice; FA – forsythoside A; HDL – high-density lipoprotein; LDL – low-density lipoprotein; MET – metformin; TC – total cholesterol; TG – triglycerides

Significant positive effects of the higher forsythoside A dose were also seen in lowering the lipid accumulation in diabetic mice (Figure 4). Again, it proved as effective as metformin. The lower forsythoside A dose was also significantly effective, but only in lowering total cholesterol and increasing HDL-C.

**Figure 5:**
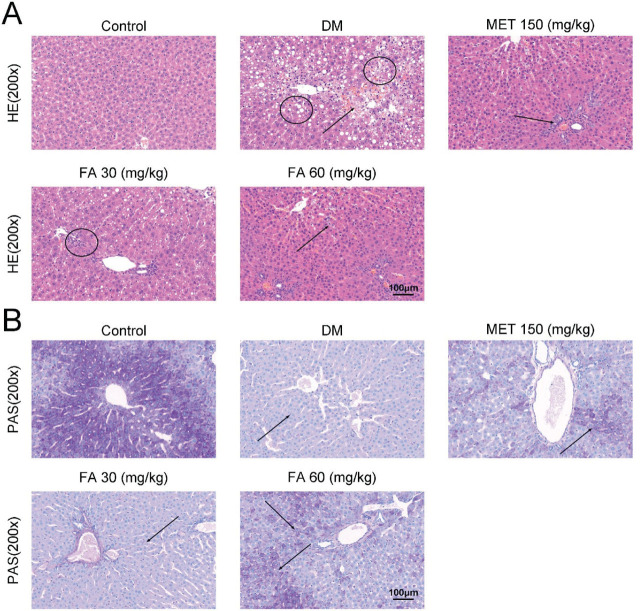
Histopathological changes in diabetic mice treated with forsythoside A vs control (200× magnification) visualised with A) HE staining showing focal necroses (circles) and mussy hepatic cords (arrows) and B) PAS staining showing hepatocyte glycogen accumulation (arrows). DM – diabetic mice; FA – forsythoside A; HE – haematoxylin and eosin; MET – metformin; PAS – periodic acid and Schiff

Histopathological examination of the liver slices revealed localised necroses and mussy hepatic cords in diabetic mice, whose occurrence was reduced by forsythoside A or metformin (Figure 5A). The two also improved liver glycogen accumulation compared to the diabetic mice (Figure 5B).

**Figure 6:**
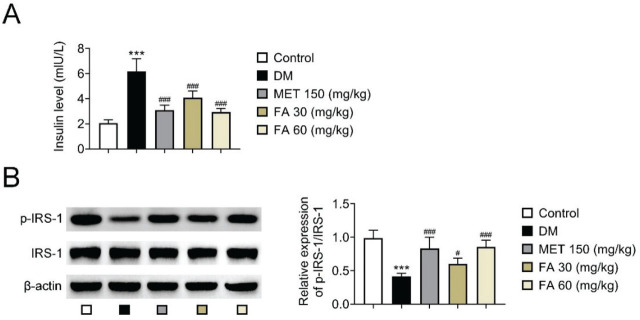
Forsythoside A-induced changes in A) serum insulin and B) p-IRS-1 expression in diabetic mice *** *p*<0.001 vs control. # *p<*0.05; ### *p*<0.001 vs DM. DM – diabetic mice; FA – forsythoside A; MET – metformin

In line with these findings, Figure 6 shows significantly lowered serum insulin levels in diabetic mice, with the higher forsythoside A dose of 60 mg/kg achieving a similar effect to metformin. At the same time, forsythoside A significantly increased p-IRS-1 protein expression, even slightly surpassing the effect of metformin at 150 mg/kg.

**Figure 7:**
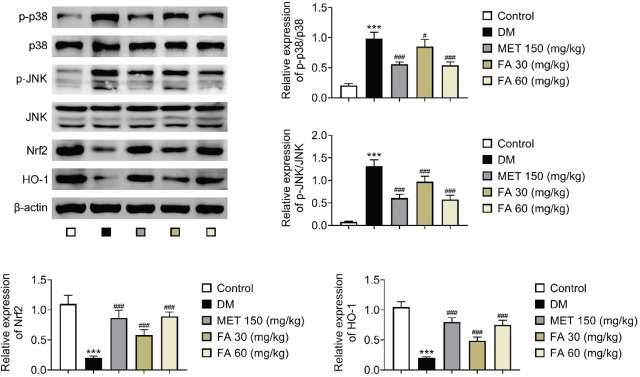
Forsythoside A-mediated changes in MAPK and Nrf2 pathways in diabetic mice. *** *p*<0.001 vs control. # *p<*0.05; ### *p*<0.001 vs DM. DM – diabetic mice; FA – forsythoside A; MET – metformin

As for its effects on the MAPK pathway, forsythoside A significantly inhibited the expression of p-p38 and p-JNK, more prominently so with the higher dose, which matched the metformin effects. In the same dose-dependent manner, it significantly increased Nrf2 and HO-1 expression matching the metformin effects (Figure 7).

Our findings consistently show that forsythoside A, the 60 mg/kg bw dose in particular, matched metformin in all antidiabetic effects without exhibiting liver toxicity. They also confirm our assumption that forsythoside A would achieve these effects through antioxidative action and that it would involve the MAPK and Nrf2 signalling pathways by inhibiting the first and promoting the second, which is in line with earlier reports (8, 9, 20,21,22).

In summary, this preliminary study demonstrated that a comparable antidiabetic effect of the 60 mg/kg forsythiaside A dose to metformin, with no obvious hepatotoxic or adverse effects. However, a complete safety profile of forsythiaside A yet remains to be established. Future studies are warranted to systematically evaluate acute and long-term toxicity, multi-organ toxicity, pharmacokinetic properties, and drug-drug interactions to determine its therapeutic window. Considering that the diabetic mouse model well reflects well the metabolic properties of human type 2 diabetes and that forsythiaside A, has a favourable safety foundation, implications for its clinical trials are promising. Future research should move towards phase I clinical trials to determine the safe dosage in humans and testing the therapeutic effect of forsythiaside A as an adjuvant to first-line antidiabetic drugs. Furthermore, in-depth investigations are needed to clarify the effects of forsythiaside A on gut microbiota and its preventive role in diabetic complications.
